# Impact of the estimation equation for GFR on population-based prevalence estimates of kidney dysfunction

**DOI:** 10.1186/s12882-017-0749-5

**Published:** 2017-11-28

**Authors:** Pietro Trocchi, Matthias Girndt, Christa Scheidt-Nave, Silke Markau, Andreas Stang

**Affiliations:** 1 Center of Clinical Epidemiology, c/o Institute of Medical Informatics, Biometry and Epidemiology (IMIBE), University Hospital Essen, Hufelandstr. 55, 45147 Essen, Germany; 2 Department of Internal Medicine II, Medical Faculty of the Martin-Luther-University Halle-Wittenberg, Ernst-Grube-Str. 40, 06120 Halle (Saale), Germany; 3 Department of Epidemiology and Health Monitoring, Robert Koch-Institute, General-Pape-Str. 62-66, 12101 Berlin, Germany; 4 Department of Epidemiology, School of Public Health, Boston University, 715 Albany St, Boston, MA 02118 USA

**Keywords:** Epidemiology, Prevalence, Renal dysfunction, eGFR equation

## Abstract

**Background:**

Estimating equations are recommended by clinical guidelines as the preferred method for assessment of glomerular filtration rate (GFR). The aim of the study was to compare population-based prevalence estimates of decreased kidney function in Germany defined by an estimated GFR (eGFR) <60 ml/min/1.73m^2^ using different equations.

**Methods:**

The study included 7001 participants of the German Health Interview and Examination Survey for Adults 2008–2011 (DEGS1) for whom GFR was estimated using the Modification of Diet in Renal Disease study equation (MDRD), the revised Lund-Malmö equation (LM), the Full Age Spectrum creatinine equation (FAScre), the Chronic Kidney Disease Epidemiology Collaboration equations with creatinine and cystatin C (CKD-EPIcrecys), with creatinine (CKD-EPIcre) and with cystatin C (CKD-EPIcys). Bland-Altman plots were used to evaluate the agreement between the equations.

**Results:**

Prevalence estimates of decreased kidney function were: 2.1% (CKD-EPIcys), 2.3% (CKD-EPIcrecys), 3.8% (CKD-EPIcre), 5.0% (MDRD), 6.0% (LM) and 6.9% (FAScre). The systematic differences between the equations were smaller by comparing either equations that include serum cystatin C or equations that include serum creatinine alone and increased considerably by increasing eGFR.

**Conclusions:**

Prevalence estimates of decreased kidney function vary considerably according to the equation used for estimating GFR. Equations that include serum cystatin C provide lower prevalence estimates if compared with equations based on serum creatinine alone. However, the analysis of the agreement between the equations according to eGFR provides evidence that the equations may be used interchangeably among persons with pronounced decreased kidney function. The study illustrates the implications of the choice of the estimating equation in an epidemiological setting.

**Electronic supplementary material:**

The online version of this article (10.1186/s12882-017-0749-5) contains supplementary material, which is available to authorized users.

## Background

Chronic Kidney Disease (CKD) is defined by morphological and functional damage to the kidney [1, 2]. Clinical assessment of kidney function is central to the routine clinical practice [3, 4] and glomerular filtration rate (GFR) is the best overall index-indicator of excretory kidney function in health and disease [3, 5]. In the epidemiological setting, decreased kidney function may be defined by a GFR <60 ml/min/1.73m^2^ [1]. As directly measuring GFR is often cumbersome in routine clinical practice, researchers have developed and validated several GFR estimating equations that include demographic and clinical variables as surrogates for muscle mass and unmeasured factors that affect serum creatinine level, such as age, sex and race. Some of these equations are meanwhile recommended by clinical guidelines as the preferred method for assessment of GFR in the routine clinical care [1].

Decreased kidney function has been shown to be an independent marker for major adverse outcomes of CKD, including progression to end-stage kidney failure and premature death caused by cardiovascular disease [6–8]**.** Patients with decreased kidney function require considerable medical attention to prevent deterioration and the development of complications. If kidney disease progresses to end-stage kidney disease, renal replacement therapy is an enormously resource consuming condition. Given its high impact on patients’ quality of life and medical resources and rising prevalence estimates reported from many countries [9], CKD is increasingly recognized a major public health problem and the knowledge of its prevalence is of great importance from both a medical and the economical standpoint. Several studies evaluated performance and limitations of different estimating equations for GFR against a gold standard of kidney function testing in a clinical setting [4, 10–14]. Although it is widely known to nephrologist that the eGFR equations perform differently in relation to patient characteristics, the behavior of the equations in unselected large population-based samples has only been investigated in detail in the NHANES study to our knowledge. Therefore, a detailed assessment of the behavior of the equations in a European, predominantly Caucasian population of 7000 participants is important for researchers who want to provide population-based prevalence estimates of kidney dysfunction. To date, literature about the prevalence of kidney function in Germany is scarce. Recently, we published population-based estimates of prevalence of kidney damage in Germany based on measures of albuminuria and the use of an established equation for GFR estimation [15]. Furthermore, the Study of Health in Pomerania (SHIP-1) and the Cooperative Health Research in the Region of Augsburg (KORA F4) reported results about prevalence of decreased kidney function in Northeast and Southern Germany respectively [16]. Finally, the Berlin Initiative Study assessed kidney function in Berlin in a cross sectional analysis of people aged 70 years and older [17]. The present study compares different population-based prevalence estimates of decreased kidney function among adults in Germany using six different GFR estimating equations.

## Methods

### Study population and design

The German Health Interview and Examination Survey for Adults (“Studie zur Gesundheit Erwachsener in Deutschland”, DEGS) is part of the health monitoring system at the Robert Koch-Institute (RKI). The concept and design of DEGS are described in detail elsewhere [18–20]. The first wave (DEGS1) was conducted from 2008 to 2011 and included interviews, examinations and tests. The DEGS1 study has a mixed design, which enables both cross-sectional and longitudinal analyses. For this purpose, a random sample from local population registries was drawn to supplement former participants from the German National Health Interview and Examination Survey 1998 (GNHIES98). To evaluate kidney function, blood samples were taken from all participants and serum creatinine concentration (Architect, Abbott Diagnostics, Wiesbaden; IDMS traceable creatinine Assay, kinetic Jaffe’s method) and serum cystatin C concentration (N Latex Cystatin C assay, Prospec, Siemens Healthcare, Eschborn) were measured. Participants with diabetes mellitus were identified according to self-reported medical history and verified current use of anti-diabetic drugs [21]. Participants with gestational diabetes were not included among those with diabetes mellitus. Arterial hypertension was assumed if the participant reported current treatment with antihypertensive medications or elevated blood pressure (≥140 mmHg systolic or ≥90 mmHg diastolic) was measured in the survey [22].

**Fig. 1:**
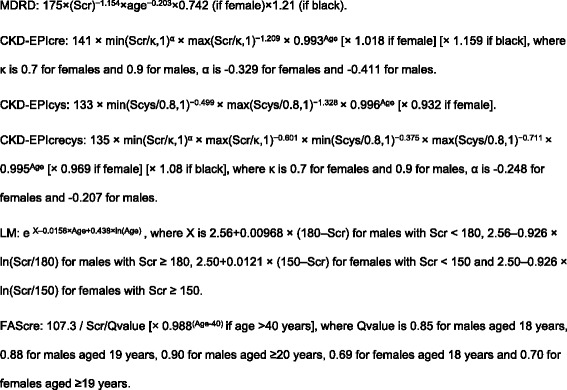
Equations used to estimate GFR. MDRD: Modification of Diet in Renal Disease study equation; CKD-EPIcre: Chronic Kidney Disease Epidemiology Collaboration creatinine equation; CKD-EPIcys: Chronic Kidney Disease Epidemiology Collaboration cystatin C equation; CKD-EPIcrecys: Chronic Kidney Disease Epidemiology Collaboration creatinine and cystatin C equation; LM: Lund-Malmö equation; FAScre: Full Age Spectrum creatinine equation; Scr: serum creatinine; Scys: serum cystatin C; min: minimum; max: maximum

The analyses presented here refer to the sample of 7001 participants of the DEGS1 aged 18–79 years for whom estimated GFR (eGFR) was calculated using six GFR estimating equations (creatinine was measured in mg/dl, cystatin C in mg/l): the isotope dilution mass spectrometry traceable Modification of Diet in Renal Disease study equation (MDRD) [23], the revised Lund-Malmö equation (LM) [12], the Full Age Spectrum creatinine equation (FAScre) [24], the Chronic Kidney Disease Epidemiology Collaboration creatinine equation (CKD-EPIcre), the Chronic Kidney Disease Epidemiology Collaboration cystatin C equation (CKD-EPIcys) and the Chronic Kidney Disease Epidemiology Collaboration creatinine and cystatin C equation (CKD-EPIcrecys) [4]. Equations are detailed in Fig. 1. Persons with missing data on eGFR were excluded (*N* = 114).

### Statistical analysis

Participants were classified into four GFR categories based on the estimated GFR values (expressed in ml/min/1.73m^2^) as follows: G1 (≥90), G2 (60 < 90), G3a (45 < 60) and G3b-G5 (<45). We estimated the prevalence and corresponding 95% confidence intervals (95% CI) of a decreased kidney function, as defined by an eGFR <60 ml/min/1.73m^2^, and the prevalence of each GFR category. Furthermore, we calculated the population based estimate of the number of persons with decreased kidney function in Germany using the population census figures of the Federal Republic of Germany in 2011. Population data were provided by the Federal Bureau of Statistic.

We evaluated the agreement between the six equations used for estimating GFR according to the approach proposed by Bland and Altman [25]. Bland-Altman plots display for each person the difference between two measurements against their mean. To compare the equations with each other, the absolute mean difference (md) between eGFR was used as a measure of the magnitude of the systematic difference (bias) between the equations. In addition, as a measure of relative change of eGFR, we calculated the mean percent difference between the measurements. The limits of the agreement between the equations were defined as md ±1.96 standard deviation of the differences (SD) and were used as a measure of the variability of the bias. These values represented the range within which 95% of the differences were included (95% CI agreement). Furthermore, for each of the ten pairwise comparisons, the analysis of the md was stratified by the GFR category based on the mean value of eGFR (G1: ≥90, G2: 60 < 90, G3a: 45 < 60, G3b-G5: <45).

Prevalence estimates were weighted by a factor that corrects sample deviations from population structure (as of 31 Dec. 2010) with regard to age, sex, region and nationality, type of community and education. When calculating the weighting factor for previous participants of GNHIES98, the probability of repeated participation, based on a multivariable logistic model, was taken into account. A non-response analysis and a comparison of selected indicators with data from official statistics indicate a high level of sample representativeness for the resident population of Germany aged 18–79 years [18]. In addition, the observed number of subjects end stage renal disease in our sample (*n* = 1) was similar to the expected number of subjects with end stage renal disease (*n* = 7) as derived from a recent report [26]. To take into account both the weighting and the correlation of the participants within a community, confidence intervals were determined using the survey procedures in SAS® (SAS Inc., Cary, NC, USA), Version 9.4.

## Results

Table 1 shows details about the main characteristics of the 7001 participants of the DEGS1 study from whom the GFR was estimated using different eqs. (3364 men and 3637 women). The median age was 46.9 years (men: 46.4, women: 47.5). The prevalence estimates of persons with medical history of arterial hypertension and diabetes mellitus were 31.5% and 6.6% respectively. Table 2 displays the estimated prevalence of participants with decreased kidney function, defined as eGFR <60 ml/min/1.73m^2^, stratified by sex and age group. Overall, the prevalence of participants aged 18–79 years with decreased kidney function differed considerably depending on the equation used and was as follows: 2.1% (CKD-EPIcys), 2.3% (CKD-EPIcrecys), 3.8% (CKD-EPIcre), 5.0% (MDRD), 6.0% (LM) and 6.9% (FAScre). The prevalence ranged from 1.6% to 5.6% among men and from 2.6% to 8.2% among women using the CKD-EPIcys equation and the FAScre equation respectively. Whatever equation was used, the estimated prevalence was higher for women than for men and increased with age in both sexes: among participants aged <60 years the prevalence varied from 0.2% to 1.2% and increased among participants aged 70 < 80 years up to 11.4% using CKD-EPIcys and up to 38.4% using FAScre. The mean eGFR varied from 83.7 using LM to 111.4 using CKD-EPIcys.

**Table 1:** Characteristics of 7001 adults aged 18–79 in Germany 2008–2011 (DEGS1)

Characteristic	Overall		Men		Women	
Sex: N, %	7001	100	3364	49.9	3637	50.1
Age (Years): median (P10, P90)	46.9 (23.6, 70.4)		46.4 (23.3, 69.8)		47.5 (23.9, 70.9)	
BMI (Kg/m^2^): median (P10, P90)	26.2 (21.0, 33.4)		26.7 (22.1, 32.9)		25.4 (20.4, 33.8)	
Serum creatinine (mg/dl): median (P10, P90)	0.82 (0.67, 1.06)		0.92 (0.76, 1.13)		0.75 (0.63, 0.90)	
Serum cystatin C (mg/l): median (P10, P90)	0.70 (0.57, 0.90)		0.73 (0.61, 0.90)		0.67 (0.55, 0.89)	
Medical history						
Hypertension: N, %	2585	31.5	1349	33.4	1236	29.7
Diabetes mellitus: N, %	539	6.6	305	7.0	234	6.2

*P10* 10th percentile, *P90* 90th percentile, *BMI* body mass index

**Table 2:** eGFR and estimated prevalence of decreased kidney function (eGFR <60 ml/min/1.73m^2^) among 7001 adults aged 18–79 in Germany 2008–2011 (DEGS1) according to the equation used

	eGFR: Mean (SD, CV)	Prevalence (95% CI)			
		Overall	<60 years	60 < 69 years	70 < 79 years
Overall		*N* = 7001	*N* = 4538	*N* = 1376	*N* = 1087
MDRD	88.4 (59.3, 0.67)	5.0 (4.3–5.7)	1.2 (0.8–1.5)	10.9 (8.8–13.1)	20.6 (17.3–23.9)
CKD-EPIcre	95.2 (40.9, 0.43)	3.8 (3.3–4.4)	0.4 (0.2–0.6)	7.8 (5.9–9.6)	19.2 (16.1–22.4)
CKD-EPIcys	111.4 (34.3, 0.31)	2.1 (1.7–2.5)	0.2 (0.1–0.3)	3.8 (2.3–5.4)	11.4 (8.9–13.9)
CKD-EPIcrecys	105.1 (34.5, 0.33)	2.3 (1.9–2.7)	0.2 (0.1–0.3)	3.8 (2.2–5.3)	12.8 (10.4–15.3)
LM	83.7 (25.3, 0.43)	6.0 (5.3–6.7)	0.5 (0.3–0.8)	11.7 (9.5–13.8)	31.4 (27.6–35.2)
FAScre	91.7 (54.8, 0.60)	6.9 (6.1–7.6)	0.5 (0.3–0.7)	11.9 (9.9–14.0)	38.4 (34.2–42.5)
Men		*N* = 3364	*N* = 2150	*N* = 667	*N* = 547
MDRD	91.4 (45.7, 0.50)	4.1 (3.3–4.8)	0.9 (0.5–1.3)	9.4 (6.4–12.4)	18.3 (14.4–22.1)
CKD-EPIcre	96.4 (32.2, 0.34)	3.6 (2.9–4.4)	0.5 (.0.2–0.8)	7.7 (4.9–10.4)	19.0 (14.9–23.1)
CKD-EPIcys	113.5 (28.1, 0.25)	1.6 (1.1–2.0)	0.2 (0.0–0.4)	2.8 (0.7–4.8)	8.6 (5.8–11.4)
CKD-EPIcrecys	106.8 (27.6, 0.26)	1.7 (1.3–2.2)	0.3 (0.1–0.5)	2.3 (0.3–4.3)	10.5 (7.6–13.4)
LM	84.0 (28.1, 0.33)	5.6 (4.8–6.5)	0.5 (0.3–0.8)	12.3 (9.1–15.5)	30.5 (25.6–35.5)
FAScre	94.2 (43.8, 0.46)	5.6 (4.7–6.4)	0.4 (0.1–0.6)	11.3 (8.2–14.4)	32.1 (27.0–37.1)
Women		*N* = 3637	*N* = 2388	*N* = 709	*N* = 540
MDRD	85.5 (43.5, 0.51)	5.9 (4.9–6.9)	1.5 (0.9–2.1)	12.4 (9.3–15.5)	22.6 (17.9–27.3)
CKD-EPIcre	94.0 (32.6, 0.35)	4.0 (3.2–4.8)	0.3 (0.1–0.6)	7.8 (5.3–10.4)	19.4 (14.9–24.0)
CKD-EPIcys	109.3 (29.6, 0.27)	2.6 (2.0–3.3)	0.1 (0.0–0.2)	4.9 (2.4–7.4)	13.8 (9.7–17.8)
CKD-EPIcrecys	103.4 (30.0, 0.29)	2.8 (2.2–3.4)	0.1 (0.0–0.1)	5.2 (2.8–7.6)	14.8 (10.9–18.7)
LM	83.4 (27.4, 0.33)	6.4 (5.4–7.3)	0.5 (0.2–0.9)	11.1 (8.2–14.0)	32.2 (27.2–37.1)
FAScre	89.3 (40.8, 0.46)	8.2 (7.1–9.2)	0.5 (0.2–0.9)	12.6 (9.7–15.5)	43.6 (38.3–49.0)

*SD* standard deviation, *CV* coefficient of variation, *CI* confidence interval, *MDRD* Modification of Diet in Renal Disease study equation, *CKD-EPIcre* Chronic Kidney Disease Epidemiology Collaboration creatinine equation, *CKD-EPIcys* Chronic Kidney Disease Epidemiology Collaboration cystatin C equation, *CKD-EPIcrecys* Chronic Kidney Disease Epidemiology Collaboration creatinine and cystatin C equation, *LM* Lund-Malmö equation, *FAScre* Full Age Spectrum creatinine equation

Based on the age specific prevalence estimates and the German population in 2011, the estimated number of persons aged 18–79 years with decreased kidney function in Germany varied from 1.41 m using the CKD-EPIcys equation to 4.58 m using the FAScre equation. Assuming that the prevalence of persons aged ≥80 with decreased kidney function equals that of the study participants aged 75 < 80 years, the total number of persons with decreased kidney function ranged from 2.15 m using the CKD-EPIcys equation to 6.78 m using the FAScre equation (Table 3).

**Table 3:** Estimated numbers (millions) of adults with decreased kidney function (eGFR <60 ml/min/1.73m^2^) in Germany 2011 according to the equation used

Age (years)	MDRD	CKD-EPIcre	CKD-EPIcys	CKD-EPIcrecys	LM	FAScre
<50	0.179	0.039	0.036	0.022	0.041	0.016
50 < 60	0.373	0.162	0.037	0.058	0.214	0.199
60 < 70	0.944	0.665	0.324	0.320	0.999	1.018
70 < 80	1.772	1.672	1.015	1.119	2.773	3.349
≥80^a^	1.067	1.070	0.742	0.737	1.930	2.202
Overall	4.335	3.608	2.154	2.256	5.958	6.784

*MDRD* Modification of Diet in Renal Disease study equation, *CKD-EPIcre* Chronic Kidney Disease Epidemiology Collaboration creatinine equation, *CKD-EPIcys* Chronic Kidney Disease Epidemiology Collaboration cystatin C equation, *CKD-EPIcrecys* Chronic Kidney Disease Epidemiology Collaboration creatinine and cystatin C equation, *LM* Lund-Malmö equation, *FAScre*: Full Age Spectrum creatinine equation

^a^By use of the estimated prevalence of study participants aged 70 < 80 years

**Fig. 2:**
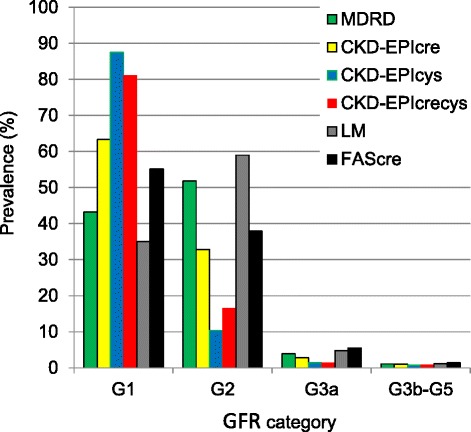
Prevalence estimates of GFR categories among 7001 adults aged 18–79 in Germany 2008–2011 (DEGS1) according to the equation used. MDRD: Modification of Diet in Renal Disease study equation; CKD-EPIcre: Chronic Kidney Disease Epidemiology Collaboration creatinine equation; CKD-EPIcys: Chronic Kidney Disease Epidemiology Collaboration cystatin C equation; CKD-EPIcrecys: Chronic Kidney Disease Epidemiology Collaboration creatinine and cystatin C equation; LM: Lund-Malmö equation; FAScre: Full Age Spectrum creatinine equation

Figure 2 presents the prevalence estimates of the GFR categories according to the equation used. Using any CKD-EPI equations, the large majority of the participants were classified as G1. In particular, according to CKD-EPIcys, almost 9 out of 10 participants were classified in this category. Using MDRD or LM the highest prevalence was estimated for the participants with eGFR 60 < 90 ml/min/1.73m^2^ (category: G2). The prevalence of participants with eGFR <45 ml/min/1.73m^2^ (category G3b-G5) varied from 0.7% using CKD-EPIcys to 1.4% using FAScre.

**Fig. 3:**
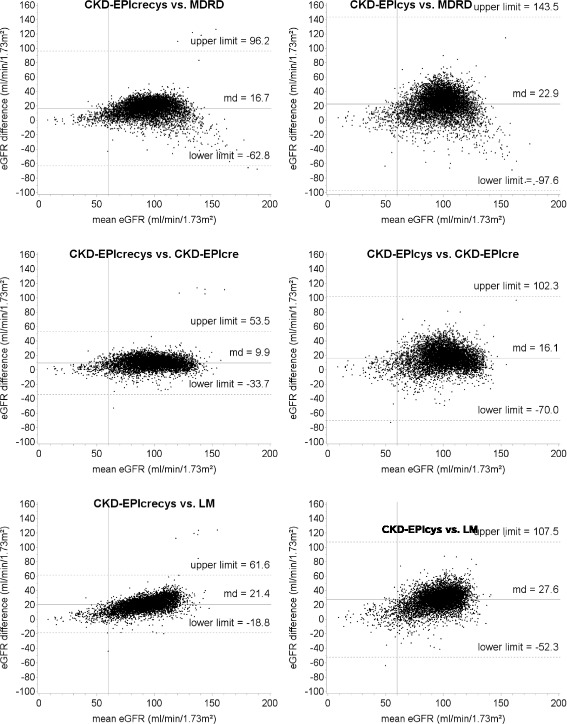
Bland-Altman plots for comparison between equations that include cystatin C and equations based on creatinine alone among 7001 adults aged 18–79 in Germany 2008–2011 (DEGS1). MDRD: Modification of Diet in Renal Disease study equation; CKD-EPIcre: Chronic Kidney Disease Epidemiology Collaboration creatinine equation; CKD-EPIcys: Chronic Kidney Disease Epidemiology Collaboration cystatin C equation; CKD-EPIcrecys: Chronic Kidney Disease Epidemiology Collaboration creatinine and cystatin C equation; LM: Lund-Malmö equation. Solid, horizontal lines represent the mean difference between the eGFR. Dashed, horizontal lines represent the limit of agreement between the equations. Solid, vertical lines represent the eGFR cut-off value for a decreased kidney function (60 ml/min/1.73m^2^)

**Fig. 4:**
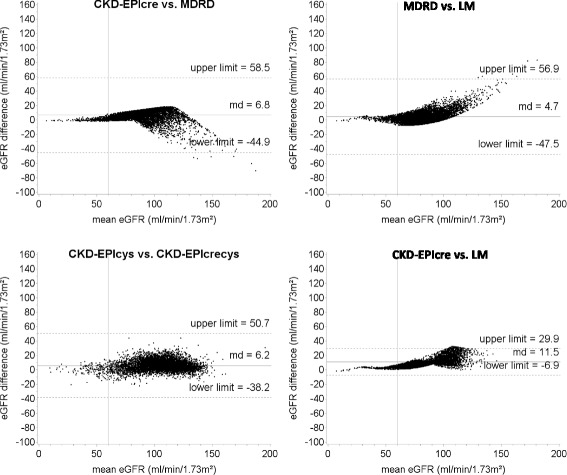
Bland-Altman plots for comparison between equations both based on cystatin C and comparison between equations both based on creatinine alone among 7001 adults aged 18–79 in Germany 2008–2011 (DEGS1). MDRD: Modification of Diet in Renal Disease study equation; CKD-EPIcre: Chronic Kidney Disease Epidemiology Collaboration creatinine equation; CKD-EPIcys: Chronic Kidney Disease Epidemiology Collaboration cystatin C equation; CKD-EPIcrecys: Chronic Kidney Disease Epidemiology Collaboration creatinine and cystatin C equation; LM: Lund-Malmö equation. Solid, horizontal lines represent the mean difference between the eGFR. Dashed, horizontal lines represent the limit of agreement between the equations. Solid, vertical lines represent the eGFR cut-off value of a decreased kidney function (60 ml/min/1.73m^2^)

Table 4 shows that great absolute differences between eGFR were calculated comparing CKD-EPIcys with LM (md = 27.6), CKD-EPIcys with MDRD (md = 22.9) and CKD-EPIcrecys with LM (md = 21.4). In contrast, small differences were calculated comparing FAScre with MDRD (md = 3.3) and CKD-EPIcre (md = 3.5), MDRD with LM (md = 4.7), CKD-EPIcys with CKD-EPIcrecys (md = 6.2) and CKD-EPIcre with MDRD (md = 6.8). Overall, the absolute md between eGFR were very small for small values of eGFR and increased considerably with increasing eGFR values. In particular, among participants with mean eGFR <45 ml/min/1.73m^2^ (G3b-G5), the md ranged from 0.1 (MDRD vs. FAScre) to 6.1 ml/min/1.73m^2^ (CKD-EPIcys vs. LM), while among participants with mean eGFR >90 ml/min/1.73m^2^ (G1) the md ranged from 2.2 (CKD-EPIcre vs. FAScre) to 30.1 ml/min/1.73m^2^ (CKD-EPIcys vs. LM). Very good levels of agreement between the equations for small values of eGFR were shown also from the Bland-Altman plots that depict the agreement over the whole range of eGFR values (Figs. 3 and 4, and Additional file 1: Figure S1). The greatest variability of the differences between eGFR, estimated as the range between the limits of the agreement, was observed comparing CKD-EPIcys with MDRD (95% CI: -97.6, 143.5).

**Table 4:** Absolute mean differences between GFR estimated by the different equations used among 7001 adults aged 18–79 in Germany 2008–2011 (DEGS1) according to GFR category

	Overall	GFR category^a^			
		G1	G2	G3a	G3b-G5
MDRD vs. CKD-EPIcrecys	16.7 (15.9%)	17.5 (15.1%)	16.1 (18.3%)	6.4 (11.1%)	2.2 (6.0%)
MDRD vs. CKD-EPIcre	6.8 (7.1%)	7.1 (6.6%)	6.9 (8.4%)	1.7 (3.1%)	0.3 (0.8%)
MDRD vs. CKD-EPIcys	22.9 (20.6%)	24.3 (20.4%)	20.0 (22.2%)	4.6 (8.2%)	3.5 (9.1%)
MDRD vs. LM	4.7 (5.6%)	9.6 (9.9%)	1.5 (1.9%)	1.4 (2.6%)	2.6 (7.7%)
MDRD vs. FAScre	3.3 (3.6%)	5.1 (4.7%)	2.0 (2.5%)	2.9 (5.5%)	0.1 (0.1%)
CKD-EPIcrecys vs. CKD-EPIcre	9.9 (10.4%)	10.1 (9.8%)	9.8 (13.2%)	5.4 (10.6%)	1.9 (5.4%)
CKD-EPIcrecys vs. CKD-EPIcys	6.2 (5.6%)	6.6 (5.7%)	4.3 (5.3%)	1.1 (2.0%)	0.8 (2.3%)
CKD-EPIcrecys vs. LM	21.4 (25.6%)	24.0 (26.1%)	17.6 (24.8%)	8.3 (16.6%)	4.8 (14.9%)
CKD-EPIcrecys vs. FAScre	13.4 (14.6%)	12.6 (12.3%)	16.2 (22.8%)	9.4 (19.0%)	2.0 (5.6%)
CKD-EPIcre vs. CKD-EPIcys	16.1 (14.5%)	16.8 (14.2%)	14.6 (16.8%)	3.5 (6.2%)	3.4 (9.0%)
CKD-EPIcre vs. LM	11.5 (13.7%)	15.0 (15.8%)	8.2 (11.0%)	3.3 (6.3%)	2.9 (8.9%)
CKD-EPIcre vs. FAScre	3.5 (3.8%)	2.2 (2.1%)	5.6 (7.4%)	4.6 (8.9%)	0.4 (1.1%)
CKD-EPIcys vs. LM	27.6 (33.0%)	30.1 (33.5%)	22.1 (32.2%)	7.0 (13.9%)	6.1 (18.7%)
CKD-EPIcys vs. FAScre	19.7 (21.4%)	19.4 (19.4%)	22.3 (32.7%)	8.0 (16.0%)	3.0 (8.7%)
LM vs. FAScre	8.0 (8.7%)	13.5 (12.4%)	3.5 (4.4%)	1.2 (2.3%)	2.1 (5.7%)

Relative changes (%) of the estimated GFR were calculated as ([first value] – [second value]) / [second value])

*MDRD* Modification of Diet in Renal Disease study equation, *CKD-EPIcre* Chronic Kidney Disease Epidemiology Collaboration creatinine equation, *CKD-EPIcys* Chronic Kidney Disease Epidemiology Collaboration cystatin C equation, *CKD-EPIcrecys* Chronic Kidney Disease Epidemiology Collaboration creatinine and cystatin C equation, *LM* Lund-Malmö equation, *FAScre* Full Age Spectrum creatinine equation

^a^GFR categories are defined according to the mean value of GFR estimated by the equations being compared (expressed in ml/min/1.73m^2^) as follows: G1: ≥90, G2: 60 < 90, G3a: 45 < 60, G3b-G5: <45

## Discussion

This study shows that prevalence estimates of decreased kidney function (eGFR <60 ml/min/1.73m^2^) among adults varies considerably depending on the equation used for estimating GFR. Prevalence estimates among of persons aged 18–79 in Germany 2008–2011 (DEGS1) varied from 2.1% using CKD-EPIcys to 6.9% using FAScre and the overall number of persons with decreased kidney function ranged accordingly from 2.15 m (CKD-EPIcys) to 6.78 m (FAScre). From a public health standpoint, the choice of the equation produces a wide range of the estimated number of persons with kidney disease in Germany.

Prevalence estimates of decreased kidney function vary substantially both within and between countries and many potential factors leading to these variations have been discussed [27–29]. Our estimated prevalence is lower than those from the SHIP-1 study and the KORA F4 study, which reported a prevalence of decreased kidney function in Northeast and Southern Germany of 5.9% and 3.1% respectively using CKD-EPIcrecys (vs. 2.3% in DEGS1) [16]. If compared with DEGS1, the median age of participants as well as the prevalence of hypertension and diabetes mellitus in these studies was considerably higher, especially for SHIP-1. Therefore, the observed differences are mostly due to differences in age and in prevalence of risk factors among the study populations. Prevalence estimates in our study were also lower than those from the US population based on the National Health and Nutrition Examination Survey (NHANES), which reported a prevalence estimate of 8% using MDRD [2]. Some reasons for these differences have been discussed in our previous publication, including heterogeneity in age distribution and ethnic characteristics of the study populations [15].

As the GFR estimating equations include the same demographic variables, such as age and sex, the observed differences between prevalence estimates could be mainly due to the fact that some equations use serum cystatin C (CKD-EPIcys, CKD-EPIcrecys), while other equations use serum creatinine alone as biomarker (MDRD, CKD-EPIcre, LM and FAScre). In particular, the prevalence of participants with decreased kidney function estimated by those equations that include serum cystatin C was considerably lower than the prevalence estimated by those equations based on serum creatinine alone. The lowest prevalence was estimated using the equation that includes cystatin C alone as laboratory parameter (CKD-EPIcys). These results are in line with published findings [30] and may reflect that there are less non-renal factors influencing cystatin C plasma levels than there are for creatinine plasma levels. Higher prevalence estimates using equations with creatinine alone were found also by the Berlin Initiative Study (BIS) for people aged 70 years and older [17]. In contrast to our study, data based on NHANES showed that equations with creatinine alone yielded lower prevalence estimates if compared with equations that included cystatin C [31]. Interestingly, in agreement with DEGS1, NHANES reported higher mean values of eGFR for equations with cystatin C if compared with mean eGFR calculated by equations with creatinine alone. Our data point out that the seemingly low level of imprecision of the creatinine based calculations may translate into quite relevant differences when using the equations for epidemiological questions and support the suggestion to use the GFR values estimated by equations with cystatin C as confirmatory test for people with decreased kidney function as estimated by equations with creatinine only [4].

Given the large difference between the prevalence estimates yielded by the different GFR estimating equations, the choice for the equation for assessing GFR can have a great impact on the assessment of public health implications, e. g. projections of disease burden or medical resources in relation to CKD. Individually, misclassification of patients as having chronic kidney disease can result in unnecessary diagnostic and therapeutic interventions with consequent added costs to the health care system. Actually, the costs of cystatin C tests vary from about 3 to 20 times those of creatinine tests [32]. It is well known that any estimating equation performs better in those populations which are alike the population in which the equation was developed. For example, the CKD-EPIcre equation was validated in a population with a majority of healthy people and therefore this equation provides accurate estimates at higher ranges of eGFR. In contrast, as the MDRD equation was developed in patients with CKD, this equation performs better in populations with lower eGFR. Finally, the BIS2 equation was explicitly designed to accurately estimate GFR in persons aged 70 years or older and should be therefore used in older populations. Therefore, in order to minimize errors in GFR estimations and to reduce the risk of misclassification the equation should be used for which the development population matches best with the population of interest. In our study, great differences between eGFR were calculated by comparing equations that include cystatin C (CKD-EPIcrecys, CKD-EPIcys) with equations based on creatinine alone (MDRD, CKD-EPIcre, LM and FAScre). However, the analysis of the agreement between the equations stratified by the mean values of eGFR shows that the absolute and relative change (percent change) of eGFR was larger among GFR categories G1 and G2 than G3b-G5. In particular, the systematic differences between eGFR among participants classified in the category G3b-G5 can be easily considered clinically not relevant. The Bland-Altman plots showed a similar distribution pattern, with good agreement between the estimating equations for low values of eGFR and increasing systematic differences with increasing eGFR. These results are consistent with those observed in other studies [33, 34] and suggest that the different estimating equations may be used interchangeably among persons with moderately to severely decreased kidney function (eGFR: <45 ml/min/1.73m^2^). Furthermore, high variability of the differences, estimated by the limits of the agreement between the equations, was observed by comparing CKD-EPIcys with equations based on creatinine alone.

This study has some limitations: First, as we did not measure the GFR by a gold standard, we could not determine which equation provides the most valid prevalence estimates of decreased kidney function for the German population. Second, information on place of residence of the study participants was not available and we could not therefore evaluate regional variability in prevalence estimates. Third, as the first wave of the DEGS study was conducted from 2008 to 2011, serum cystatin C concentration was measured using a not standardized assay which complicates the comparison with studies that used a standardized assay for cystatin C. A further limitation of our and other cross-sectional studies is the lack of a second GFR estimation after 3 months which most likely results in a false positive prevalence of kidney dysfunction.

## Conclusions

Our study illustrates the importance of the choice of the GFR estimating equation from an epidemiological point of view. Prevalence estimates of decreased kidney function in Germany are highly related to the equation used. In particular, the equations that include serum cystatin C provide lower prevalence estimates if compared with those based on serum creatinine alone. However, the analysis of the systematic differences between the eGFR suggests that the equations could be used interchangeably among persons with pronounced decreased kidney function. Additional longitudinal epidemiological studies are needed to investigate which of the available equations are most useful for prediction of CKD and associated complications at the population level.

## Additional file

Additional file 1: Figure S1. Bland-Altman plots for comparison between Full Age Spectrum creatinine equation (FAScre) and the other equations used to estimate GFR among 7001 adults aged 18–79 in Germany 2008–2011 (DEGS1). MDRD: Modification of Diet in Renal Disease study equation; CKD-EPIcre: Chronic Kidney Disease Epidemiology Collaboration creatinine equation; CKD-EPIcys: Chronic Kidney Disease Epidemiology Collaboration cystatin C equation; CKD-EPIcrecys: Chronic Kidney Disease Epidemiology Collaboration creatinine and cystatin C equation; LM: Lund-Malmö equation; FAScre: Full Age Spectrum creatinine equation. Solid, horizontal lines represent the mean difference between the eGFR. Dashed, horizontal lines represent the limit of agreement between the equations. Solid, vertical lines represent the eGFR cut-off value of a decreased kidney function (60 ml/min/1.73m^2^). (PDF 422 kb)

## Abbreviations

CI: Confidence interval
CKD: Chronic Kidney Disease
CKD-EPIcre: Chronic Kidney Disease Epidemiology Collaboration creatinine equation
CKD-EPIcrecys: Chronic Kidney Disease Epidemiology Collaboration creatinine and cystatin C equation
CKD-EPIcys: Chronic Kidney Disease Epidemiology Collaboration cystatin C eq.
CV: Coefficient of variation
DEGS: German Health Interview and Examination Survey for Adults
DEGS1: German Health Interview and Examination Survey for Adults, first wave
eGFR: estimated glomerular filtration rate
FAScre: Full Age Spectrum creatinine equation
GFR: Glomerular filtration rate
GNHIES98: German National Health Interview and Examination Survey 1998
Hg: Mercury
IDMS: Isotope Dilution Mass Spectrometry
LM: Lund-Malmö equation
max: Maximum
md: Mean difference
MDRD: Modification of Diet in Renal Disease study equation
min: Minimum
RKI: Robert Koch-Institute
Scr: Serum creatinine
Scys: Serum cystatin C
SD: Standard deviation
