# Supplementary figures and images for: Impact of the estimation equation for GFR on population-based prevalence estimates of kidney dysfunction
Source: BMC Nephrol. 2017 Nov 28;18:341. doi: 10.1186/s12882-017-0749-5 (PMC5706394; doi:10.1186/s12882-017-0749-5)

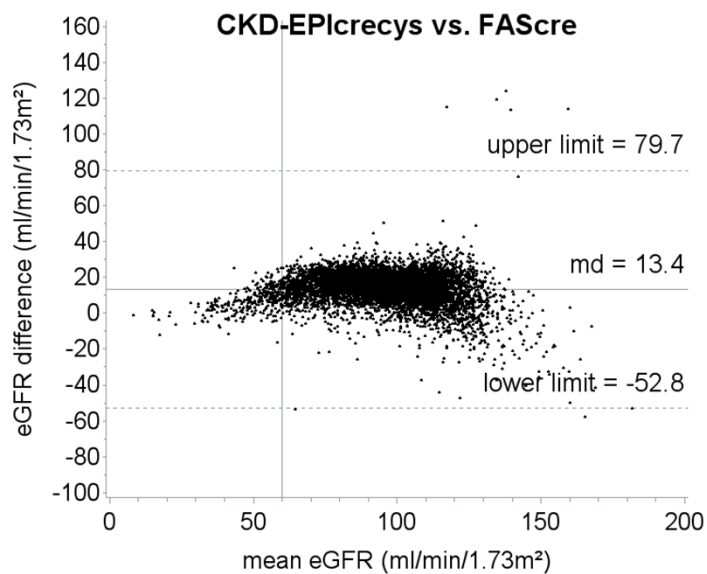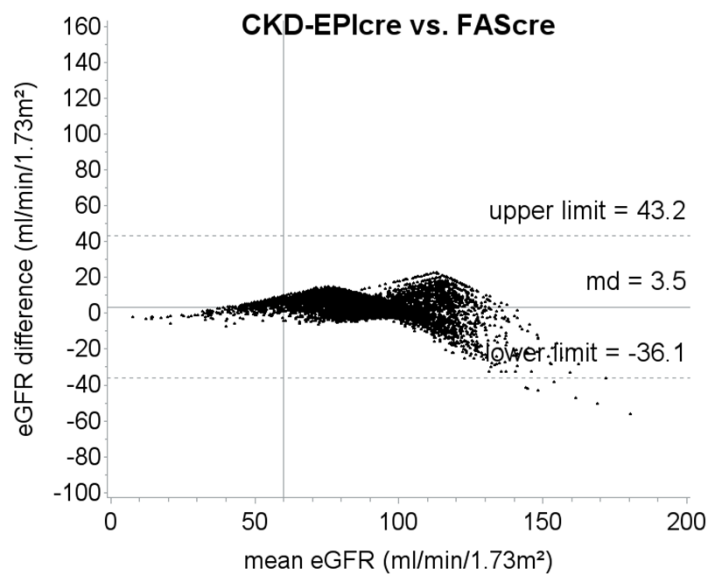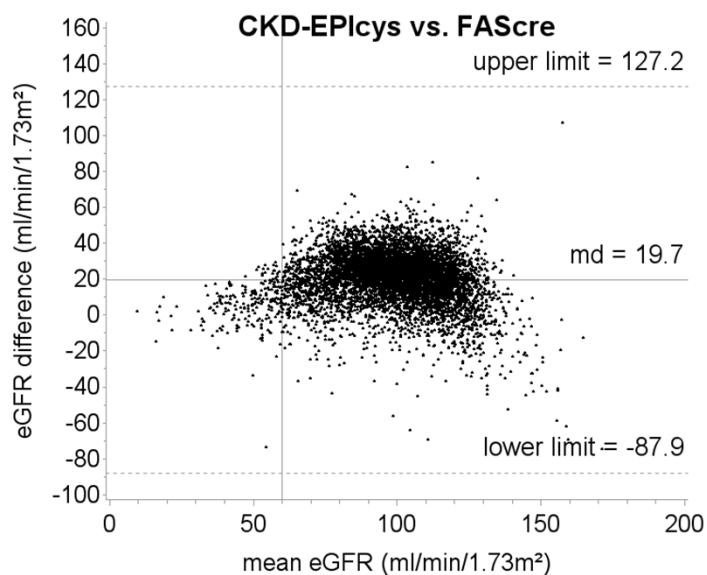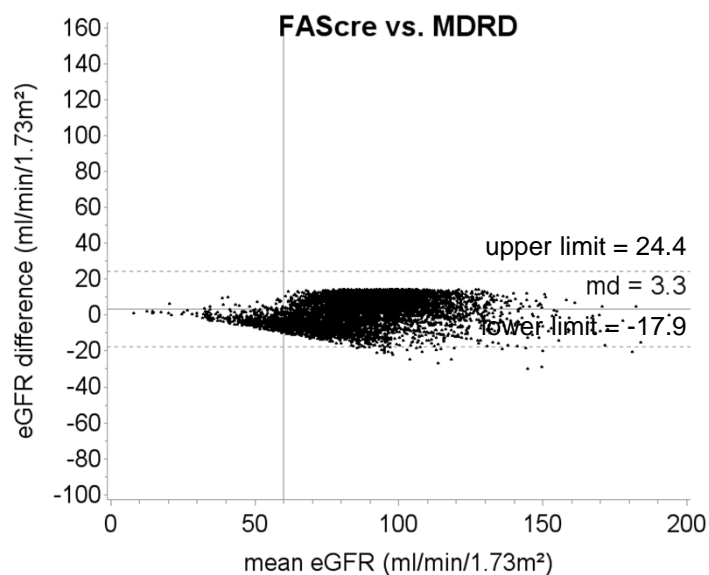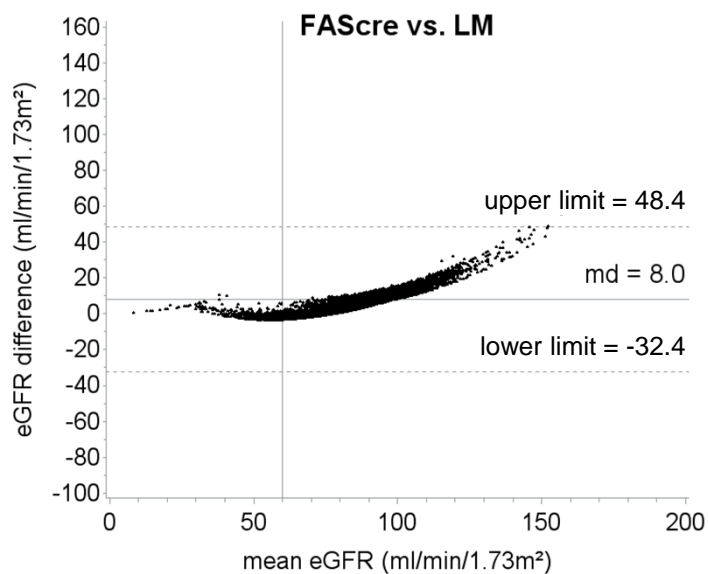

Supplement: Additional file 1: Figure S1. — Bland-Altman plots for comparison between Full Age Spectrum creatinine equation (FAScre) and the other equations used to estimate GFR among 7001 adults aged 18–79 in Germany 2008–2011 (DEGS1). MDRD: Modification of Diet in Renal Disease study equation; CKD-EPIcre: Chronic Kidney Disease Epidemiology Collaboration creatinine equation; CKD-EPIcys: Chronic Kidney Disease Epidemiology Collaboration cystatin C equation; CKD-EPIcrecys: Chronic Kidney Disease Epidemiology Collaboration creatinine and cystatin C equation; LM: Lund-Malmö equation; FAScre: Full Age Spectrum creatinine equation. Solid, horizontal lines represent the mean difference between the eGFR. Dashed, horizontal lines represent the limit of agreement between the equations. Solid, vertical lines represent the eGFR cut-off value of a decreased kidney function (60 ml/min/1.73m2). (PDF 422 kb) [file 12882_2017_749_MOESM1_ESM.pdf]
